# Proteomics to Identify Proteins Interacting with P2X2 Ligand-Gated Cation Channels

**DOI:** 10.3791/1178

**Published:** 2009-05-18

**Authors:** Harpreet Singh, Sarah Warburton, Thomas M. Vondriska, Baljit S. Khakh

**Affiliations:** Department of Physiology, David Geffen School of Medicine, University of California, Los Angeles; Department of Anesthesiology, David Geffen School of Medicine, University of California, Los Angeles; Department of Anesthesiology, Medicine and Physiology, David Geffen School of Medicine, University of California, Los Angeles

## Abstract

Ligand-gated ion channels underlie synaptic communication in the nervous system^1^. In mammals there are three families of ligand-gated channels: the cys loop, the glutamate-gated and the P2X receptor channels^2^. In each case binding of transmitter leads to the opening of a pore through which ions flow down their electrochemical gradients. Many ligand-gated channels are also permeable to calcium ions^3, 4^, which have downstream signaling roles^5^ (e.g. gene regulation) that may exceed the duration of channel opening. Thus ligand-gated channels can signal over broad time scales ranging from a few milliseconds to days. Given these important roles it is necessary to understand how ligand-gated ion channels themselves are regulated by proteins, and how these proteins may tune signaling. Recent studies suggest that many, if not all, channels may be part of protein signaling complexes^6^. In this article we explain how to identify the proteins that bind to the C-terminal aspects of the P2X2 receptor cytosolic domain.

P2X receptors are ATP-gated cation channels and consist of seven subunits (P2X1-P2X7).  P2X receptors are widely expressed in the brain, where they mediate excitatory synaptic transmission and presynaptic facilitation of neurotransmitter release^7^. P2X receptors are found in excitable and non-excitable cells and mediate key roles in neuronal signaling, inflammation and cardiovascular function^8^. P2X2 receptors are abundant in the nervous system^9^ and are the focus of this study. Each P2X subunit is thought to possess two membrane spanning segments (TM1 & TM2) separated by an extracellular region^7^ and intracellular N and C termini (Fig 1a)^7^.  P2X subunits^10^ (P2X1-P2X7) show 30-50% sequence homology at the amino acid level^11^. P2X receptors contain only three subunits, which is the simplest stoichiometry among ionotropic receptors. The P2X2 C-terminus consists of 120 amino acids (Fig 1b) and contains several protein docking consensus sites, supporting the hypothesis that P2X2 receptor may be part of signaling complexes. However, although several functions have been attributed to the C-terminus of P2X2 receptors^9^ no study has described the molecular partners that couple to the intracellular side of this protein via the full length C-terminus. In this methods paper we describe a proteomic approach to identify the proteins which interact with the full length C-terminus of P2X2 receptors.

**Figure:**
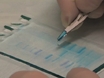


## Protocol

### EXPERIMENTAL PROCEDURES

The experimental procedure (Fig 2) consists of four parts that are described in a step-wise manner below.

### Part 1: Subcloning and expression of the C-terminus of P2X2 receptors.

We have expressed the full length C-terminus of P2X2 receptors in bacteria to identify the brain proteins to which it binds.

The C-terminus (residues 353-472) of the P2X2 receptor (Fig 1) was amplified by PCR, cloned into pGEX 4NT1 (GE Life Sciences) and verified with sequencing.
The recombinant plasmid was transformed into *E. Coli (BL21)* for expression of recombinant proteins.
Glycerol stocks of bacteria (*E. coli* BL21) containing the P2X2 CT-GST plasmid were scraped with a sterile pipette tip and inoculated into 5 ml of Luria-Bertani (LB) media with suitable selective marker (50 µg/ml ampicillin) and incubated overnight in an orbital shaker at 37°C.
The cultures grown overnight were inoculated into 250 ml of LB media with a suitable antibiotic (50 μg/ml ampicillin) and incubated in an orbital shaker at 250 rpm for 2-3 hrs at 37°C until the optical density at 600 nm reached ~ 0.6-0.7.
The culture was induced with 1 mM of IPTG and further incubated at 37°C for 3 hrs for protein expression.
The culture was then spun at 5000 g for 15 min at 4°C (1ml of culture was saved for analysing the expression level and labelled as ‘induced’). Supernatant was discarded and the pellet was resuspended in 20 ml of Saline Tris-EDTA (STE) buffer (10 mM Tris-HCl, 150 mM NaCl, 1 mM EDTA pH 8.0).
The suspension was spun at 5000 g for 15 min in a 50 ml falcon tube. The supernatant was discarded and the semi-dry pellet was frozen at -70°C overnight.
The -70°C pellet was resuspended in 40 ml of ice cold lysis buffer (2% (w/v) sarkosyl, 15 mg lysozyme, 150 mM NaCl, 50 mM Tris pH7.5, 5 mM DTT and complete protease inhibitor tablet), and the suspension was incubated on ice for 30 min. During this incubation, 3 ml Glutathione–Sepharose 4B beads were washed with 20 ml of Phosphate Buffer Saline (PBS) and 20 ml T-PBS (0.1% (v/v) tritonX-100 in PBS) followed by PBS. The beads were resuspended in 1 ml PBS.
The bacterial suspension was freeze-thawed 3 times in liquid nitrogen and sonicated (1s on/off, 30 microns for 3 min on ice) and further incubated for 30 mins with 4% (v/v) triton X-100, 10 mM MgSO_4_ and 2 mM ATP on ice.
The lysate was spun at 20000 g for 15 min at 4°C and pellet was discarded (a sample from pellet and 1 ml of supernatant was saved for SDS page to test the amounts of proteins in cytosol and membranes). The supernatant was incubated with the beads for 1 hr (or overnight) at 4°C.
The beads were spun at 500 g for 5 min and the supernatant was discarded (sample was saved for unbound fraction). The beads were washed with T-PBS five times (washes were saved for wash controls).
The beads were then resuspended in PBS to give 50% (w/v) slurry and stored in fridge at 4°C until use. The protein concentration was estimated by Bradford assay (per manufacturer’s instructions).

### Part 2: Preparation of the whole brain lysates

P2X2 receptors are known to be abundantly expressed in the brain^8^. In the present analyses, we sought to identify the proteins interacting with P2X2 receptors from rat whole brain lysates (Fig 3).

Adult rat brain was harvested (animals were sacrificed according to a UCLA IAACUC-approved protocol and according to the NIH Guide for the Care and Use of Laboratory Animals) and rinsed immediately ice cold PBS (3X).
To lyse the cells 10 ml (~5 times the wet weight of harvested brain) of ice cold lysis buffer containing 150 mM NaCl, 50 mM Tris-HCl, pH 7.4, 10 mM EDTA, 1 mM EGTA, 1 mM NaF, 1 mM Na_3_VO_4_, 1 mM PMSF, 5 μg/ml of leupeptin, 1% (v/v) NP-40, and a protease inhibitor cocktail tablet was added to the harvested brain.
Brain tissue was homogenised on ice with a glass Dounce homogenizer until a mixture of homogeneous consistency was achieved.
Cell lysate was spun at 2000 g for 5 minutes to remove the unbroken cells. The supernatant was collected and spun again at 29000 g for 60 minutes to remove intact organelles and membrane aggregates.
Immediately after centrifugation the supernatant was transferred to a clean tube. The protein concentration of the whole brain lysate was measured by Bradford assay (usually ~15 mg/ml).
The lysate was aliquoted and stored at -70°C.

### Part 3: Isolation of P2X2 C-tail associated proteins

To identify the binding partners of P2X2 CT-GST from whole brain lysates, a pull down assay was performed by using CT-GST immobilised on glutathione sepharose 4B beads as bait. For controls, GST alone bound to beads was used.

Brain lysate was precleared with GST beads for 1 hr at 4°C. The pellet was discarded and the supernatant was used for further analyses.
Equal amounts of GST and P2X2 CT-GST (10 μg) bound to glutathione sepharose 4 B beads were incubated overnight with 100 µg of precleared solubilised proteins from rat whole brain lysate, with rotation at 4°C in the lysis buffer.
Beads were spun at 1000 g for 5 min and the supernatant was discarded.
The beads were washed once with the lysis buffer and boiled in modified Laemli buffer [4% (w/v) SDS, 10% (v/v) 2-mercaptoethanol, 2% (v/v) glycerol, 0.004% (w/v) bromophenol blue and 0.125 M TrisCl pH 6.8] for 5 min.
The samples were spun at 5000 g for 5 min and the supernatant separated by 10% SDS-PAGE (Fig 4). Gels were stained with SYPRO® Ruby protein gel stain (according to the manufacturer’s instructions) and visualized under ultraviolet light. P2X2 CT-associated proteins were compared with those recovered using GST-null as bait. Four biological replicates were performed (i.e. four brains and four independent pull downs).

### Part 4: Identification of proteins.

Proteins separated by gel electrophoresis were excised from the gel and identified by mass spectrometry^12^.  *Note: absence of dust throughout the process is critical to reduce keratin contamination. The experimenter should wear a face mask, hair net and gloves at all times and never touch the gel region of interest without the use of gloves.*

All visible protein bands recovered from the P2X2 pull down were excised with a clean razor blade and diced (Fig 4). Corresponding regions of the gel in the GST-null pull down lane were also excised in the same manner without regard to band staining (bands specific for the GST-null bait were also excised and identified) to insure that presence of proteins below the level of staining sensitivity was not missed.
The gel pieces were incubated with 200 μl of 50 mM ammonium bicarbonate in 50% (v/v) acetonitrile (ACN) and the tubes vortexed for 15 min at RT. Repeat this wash step.
The gel pieces were dehydrated by adding 200 μl acetonitrile (gel pieces should shrink and become opaque in colour).
Acetontrile was removed and the gel pieces dried in a speedvac for ~10 min.
The gel pieces were then incubated with 30 μl of the freshly prepared 10 mM DTT/10 mM Tris (2-carboxy-ethyl) phosphine hydrochloride (TCEP) of sufficient volume to immerse the pieces. This was left for 30 min at 56°C water bath.
Next the DTT solution was replaced with 100 μl 500 mM ammonium bicarbonate in 50% acetonitrile solution and the gel slices washed with ammounium bicarbonate.
The washing solution was aspirated and 200 μl of acetonitrile added to dehydrate the gels.
Acetonitrile was then removed and 100 μl freshly prepared 100 mM iodoacetamide solution was added to alkylate free sulfhydryls. This step proceeded for 25 min at room temperature in the dark.
Iodoacetamide solution was removed and the gel pieces were washed with 200 μl of 50 mM ammonium bicarbonate in 50% acetontrile (pH 8.0) for 2 min while vortexing. This wash step was repeated.
After removing the wash solution, the gel pieces were incubated with 200 μl acetonitrile which was then removed by speedvac for ~10min.
At this step the gel pieces are ready for trypsin digestion. Gel pieces were incubated in 30 μl trypsin solution (20 ng/μL working concentration) and placed on ice for 10 min for the gels to be well swollen. Excess trypsin was removed and rehydrated gel particles overlaid with 30 μl with 50 mM ammonium bicarbonate to keep them immersed throughout digestion which was allowed to proceed for 12 to 16 hours at 37°C.
Five μl of 5% (v/v) Formic acid is added to deactivate the trypsin and arrest digestion.
Tubes were vortexed for ~15 min and centrifuged briefly to bring the liquid to the bottom of the tube which was then transferred to a clean and labeled 0.5 ml vial.
Next 30 μl of 0.1% (v/v) Formic acid in 50% acetonitrile was added to gel pieces so they were just covered followed by vortexing twice for 10-15 min, separated by brief centrifugation. This step was repeated once, replacing the formic acid-acetonitrile between steps.
The final liquid was removed and all extraction solution combined in a single vial. This volume was reduced to ~30 uL in the speedvac. The sample was now ready for reverse phase nano-liquid chromatography and protein identification by mass spectrometry. In this specific example, this step is carried out on an Orbi-trap tandem mass spectrometer from ThermoFisher (chosen for its high mass accuracy, fast duty cycle for protein detection and generally robust operating features), although other models and manufacturers’ instruments could easily be coupled with the approach described to this point.
The details and criteria for mass spectrometric analyses are now briefly presented. All peptides were separated by reverse phase nano-LC (Eksigent) and peptides were loaded onto a C18 reverse-phase column at a flow rate of 3 μl/min. Mobile phase A was 0.1% formic acid and 2% ACN in water; mobile phase B was 0.1% formic acid and 20% water in ACN. Peptides were eluted from the column at a flow rate of 220 nl/min using a linear gradient from 5% B to 50% B over 90 min, then to 95% B over 5 min, and finally keeping constant 95% B for 5 min. Spectra were acquired in data-dependent mode with the Orbi-trap used for MS scans and LTQ for MS/MS scans. Peptides were identified by searching the spectra against the rat IPI database v.3.53 using the SEQUEST algorithm integrated into the Bioworks software package. Each peptide met the following criteria: XCorr ≥2 (+1), ≥3 (+2), ≥4 (+3), and DeltaCN>0.1. All spectra used for protein identification were manually inspected and no proteins accepted when less than two peptides were identified. Only proteins present following each of the four P2X2 pull downs and absent during GST-null pull downs were considered for further analyses.

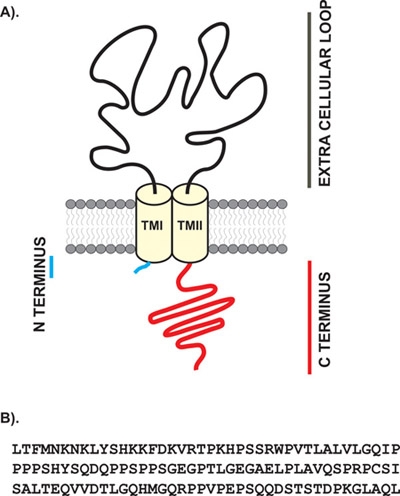


**Figure 1. Schematic representation of a P2X2 receptor subunit. A.** The cartoon illustrates the topology of a P2X2 receptor subunit. The cytosolic domain consists of N and C termini. The C-terminus of P2X2 receptor (red) was used as bait for pull down assay. **B.** Amino acid sequence of the P2X2 receptor C-terminus used in this study.


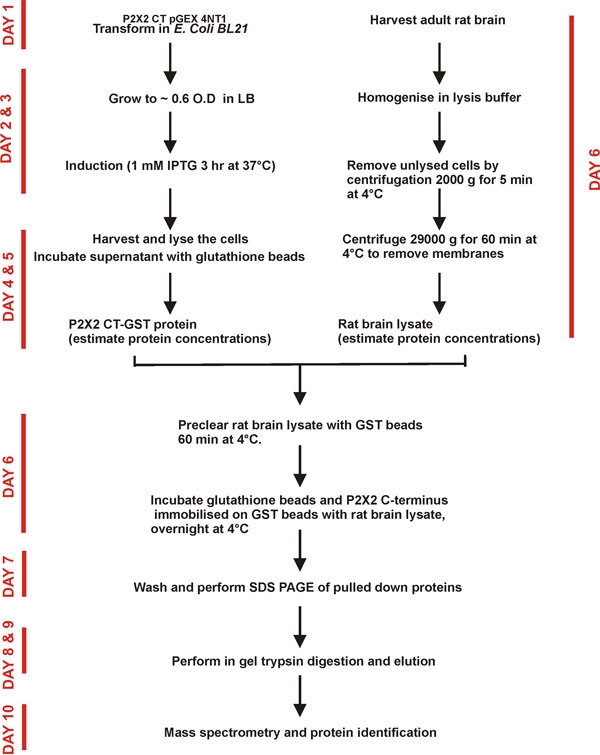


**Figure 2. Flow chart and time line of the protocol used for expression, purification, pull down and identification of proteins.** We show the outline of the protocol with the time line. Adult rat brain lysate was prepared fresh immediately before use for the pull down assays.


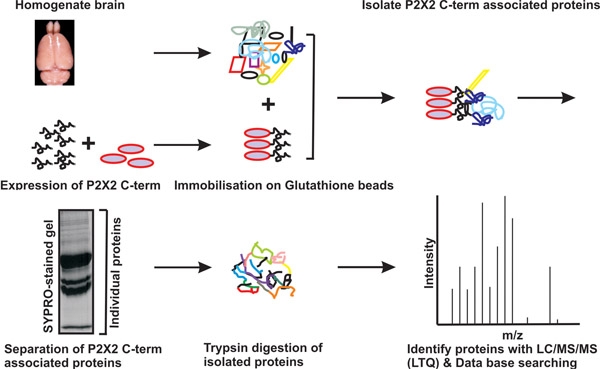


**Figure 3. Schematic representation of the binary proteomic analysis of P2X2 C-terminus network in the rat brain. ** The C-terminus of P2X2 receptors fused with GST protein bound to GST beads was used for pull down assays. Brain lysate was prepared and proteins were incubated with the immobilized recombinant proteins. Unbound fraction was washed with the lysis buffer. Proteins were identified by mass spectrometry.


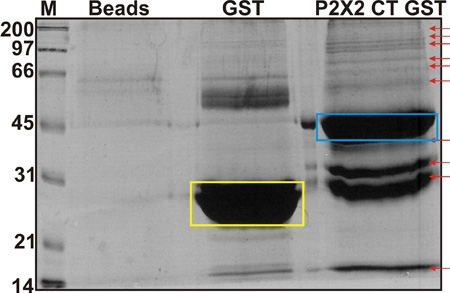


**Figure 4.  Identification of binding partners of the C-terminus of P2X2 receptors.** Sypro stained gel showing a spectrum of putative proteins that interact with the C-terminus of receptors fused with GST (blue box). Controls lanes for GST alone (yellow box) and glutathione sepharose beads alone are also shown. The arrows indicate examples of unique bands that were excised for further analysis by mass spectrometry.

## Discussion

Ion channels are a major class of integral membrane proteins. They contain water filled pores that selectively permit the movement of ions down their electrochemical gradients across the plasma membrane. Ion channels gate between open and the closed states. The gating step is triggered by transmitters (e.g. ATP) in case of P2X ligand gated ion channels, or it may be regulated by interactions with other proteins. The last decade has witnessed an increase in our understanding of how P2X receptors bind ATP^13^, but the role of ancillary proteins in regulating P2X function remains less well understood. The approach described in this protocol is designed to identify the signaling complexes formed by P2X2 receptors by examining (as a first step) the proteins interacting with the intracellular C-terminus. A complete map of the P2X2 receptor signaling complex will provide much needed insight into the pathophysiology of these fascinating channels.
